# Effect of Chemical Vapor Deposition WS_2_ on Viability and Differentiation of SH-SY5Y Cells

**DOI:** 10.3389/fnins.2020.592502

**Published:** 2020-10-30

**Authors:** Domenica Convertino, Neeraj Mishra, Laura Marchetti, Mariantonietta Calvello, Alessandro Viegi, Antonino Cattaneo, Filippo Fabbri, Camilla Coletti

**Affiliations:** ^1^National Enterprise for nanoScience and nanoTechnology Laboratory, Scuola Normale Superiore, Pisa, Italy; ^2^Center for Nanotechnology Innovation @NEST, Istituto Italiano di Tecnologia, Pisa, Italy; ^3^Department of Pharmacy, University of Pisa, Pisa, Italy; ^4^BIO@SNS Laboratory, Scuola Normale Superiore, Pisa, Italy; ^5^NEST Istituto Nanoscienze—CNR and Scuola Normale Superiore, Pisa, Italy

**Keywords:** transition metal dichalcogenides, WS_2_, graphene, sapphire, SH-SY5Y cells, differentiation

## Abstract

In recent years, transition metal dichalcogenides have been attracting an increasing interest in the biomedical field, thus implying the need of a deeper understanding of their impact on cell behavior. In this study we investigate tungsten disulfide (WS_2_) grown via chemical vapor deposition (CVD) on a transparent substrate (sapphire) as a platform for neural-like cell culture. We culture SH-SY5Y human neuroblastoma cells on WS_2_, using graphene, sapphire and standard culture well as controls. The quality, thickness and homogeneity of the materials is analyzed using atomic force microscopy and Raman spectroscopy. The cytocompatibility of CVD WS_2_ is investigated for the first time by cell viability and differentiation assessment on SH-SY5Y cells. We find that cells differentiated on WS_2_, displaying a viability and neurite length comparable with the controls. These findings shine light on the possibility of using WS_2_ as a cytocompatible material for interfacing neural cells.

## Introduction

Two-dimensional materials have shown superior properties as platform for neural cell culture. Several works have demonstrated that neural cells grown on graphene have peculiar properties improving neuronal firing (Pampaloni et al., 2018), neural stem cell differentiation (Park et al., 2011), network formation (Tang et al., 2013), and neurite sprouting (Li et al., 2011; Convertino et al., 2018, 2020). It has been reported that neurons exposed to graphene nanosheets dispersion showed altered neural signaling (Bramini et al., 2016; Rauti et al., 2016), while the use of planar graphene-based materials did not affect neuronal network function and formation (Fabbro et al., 2016).

In light of these findings highlighting the promising role of graphene in biomedical devices, in recent times also the class of two-dimensional materials known as transition metal dichalcogenides (TMDs), has gained interest for its possible adoption in biological and/or biomedical environments (Wang et al., 2012). Transition metal dichalcogenides, especially in their exfoliated flake form, have been used in bioimaging, biosensing, as antimicrobial agents, and to realize nanocomposites for tissue engineering applications to promote cell adhesion and improve mechanical properties of the scaffolds (Kalantar-zadeh et al., 2015; Agarwal and Chatterjee, 2018; Zhou et al., 2020). Tungsten disulfide (WS_2_) is one of the most studied TMD materials, thanks to its tribological, optical and electrical properties (Ovchinnikov et al., 2014; Büch et al., 2018; Cong et al., 2018). Tungsten disulfide has been used as lubricant in orthodontia (Redlich et al., 2008), or to improve the mechanical properties of biodegradable polymers in bone tissue engineering (Lalwani et al., 2013). Moreover WS_2_ has been applied in photothermal and photodynamic therapy, thanks to its good water solubility and high near infrared absorption capability (Chimene et al., 2015). Thanks to its known photoelectric characteristics (Rossi et al., 2018; Wang et al., 2019), WS_2_ could be used to realize a stimulating electrode based on the local photocurrent controlled by light, similarly to the light-directed electrical stimulation reported in the past for silicon wafer (Starovoytov et al., 2005). Due to this increasing interest, WS_2_ biocompatibility and toxicity tests need to be performed. By testing different exfoliated TMDs, Teo et al. demonstrated that the chalcogen plays an important role in influencing the material toxicity. In particular, by exposing cells to different concentrations of TMDs, they demonstrated that WS_2_ were much less toxic than graphene oxide, while WSe_2_ showed similar toxicity (Teo et al., 2014). Suhito at al. used a low concentration of WS_2_ flakes (≤1 μg/ml) to coat glass substrates, showing an improved cell adhesion, spreading, and proliferation when compared to the non-treated substrates (Suhito et al., 2017). A low cytotoxicity and genotoxicity was also reported for WS_2_ large flakes onto polydimethylsiloxane (PDMS; Appel et al., 2016). Despite the increased interest, only one work has investigated to date the effect of WS_2_ grown via chemical vapor deposition (CVD), an ideal scalable substrate to culture cells and possibly develop biomedical and biosensing devices, on cells (Palumbo et al., 2018). This study examined the morphology of fibroblast cells on islands of monolayer (ML) CVD WS_2_ grown on SiO_2_, only after transferring onto PDMS, finding an improved cell adhesion and elongation with respect to SiO_2_ and PDMS (Palumbo et al., 2018). To date, there is no study assessing the viability of cells cultured on uniformly CVD-grown WS_2_ on growth substrate with respect to standard culture substrates such as culture wells.

In this work, we evaluate CVD WS_2_ as a platform to culture neuronal-like cells and compare its performance with respect to the most studied graphene and to standard controls. We chose a transfer-free approach to ensure cleanliness and reduce contaminants. We adopt sapphire to synthesize WS_2_ and graphene, as its transparency allows to directly probe cell-substrate interaction with a label-free approach using standard transmitted light microscopy. Sapphire is a single-crystal aluminum oxide (Al_2_O_3_) that has a broad transmission band from UV to near-infrared (Kurlov, 2016). Its chemical, thermal, electrical, mechanical and optical properties make it an attractive material for a variety of applications, ranging from optoelectronics to semiconductor industry (Kurlov, 2016). Thanks to its inertness to biological tissues and biocompatibility, in the past years it has been suggested for different medical application (Shikunova and Kurlov, 2016), including bone, dental and neural implants (Khan et al., 2011; Sharova et al., 2018). In the past years, sapphire has been used to grow scalable and high-quality WS_2_ (Zhang et al., 2013; Lan et al., 2018) and graphene (Fanton et al., 2011; Hwang et al., 2013; Mishra et al., 2019). For these reasons, together with the possibility to grow 2D materials directly on it without the need to transfer them, sapphire represents an ideal substrate material for the biocompatibility investigation of 2D materials. We choose for our studies the SH-SY5Y neuroblastoma cell line since it is a well-established cellular model for experimental neurobiology studies, including metabolism, neural differentiation and neurodegenerative processes (Encinas et al., 2002). In fact, the sequential treatment of these cells with retinoic acid (RA) and human brain−derived neurotrophic factor (hBDNF), causes differentiation in a neuron-like phenotype with neuritic processes (Hromadkova et al., 2020). High quality full-covered WS_2_ and graphene samples were grown on sapphire via CVD, and bare sapphire substrates and culture wells were used as controls to test the material effect on cell viability and differentiation. We first checked the quality and homogeneity of the synthesized materials by using atomic force microscopy (AFM) and Raman spectroscopy. We then used the bare substrates (i.e., without polymeric coating) to culture SH-SY5Y. Cell viability was assessed in the WST-8 cell proliferation assay and cell differentiation was investigated via optical microscopy, which allowed quantifying the morphometric parameters that describe the differentiation process.

## Materials and Methods

### Samples Preparation and Characterization

Sapphire dice were cut from single crystal HEMCOR, double side polished sapphire (0001) wafers in square dice of about 6 mm × 6 mm. Prior to growth of graphene and WS_2_, the sapphire substrates were cleaned with acetone, isopropanol, and de-ionized (DI) water for 5 min each respectively in ultra-sonic bath. The substrates were then cleaned in piranha solution (1:3, H_2_O_2_:H_2_SO_4_) for 15 min, sonicated in DI water and finally dried with N_2_. Cleaned sapphire dice were used as control samples.

Tungsten disulfide was grown directly on sapphire via a low-pressure CVD process similar to that reported in (Rossi et al., 2016). Specifically, tungsten trioxide WO_3_ (Sigma Aldrich, 99.995%) and sulfur S (Sigma Aldrich, 99.998%) were used as solid precursors. The reactor comprises a central hot-zone, where a crucible loaded with WO_3_ powder and an inlet zone, where the S powder was positioned and heated by a resistive belt. Sulfur was heated at 100°C. The sulfur vapor reacts with WO_3_ directly on the substrate surface at a temperature of 930 °C and at a pressure of ∼5 × 10^–2^ mbar with argon (Ar) as carrier gas. In order to obtain a homogeneous coverage over the entire sample (6 mm × 6 mm) the growth process was repeated twice. The first growth process allowed for the nucleation of micrometric WS_2_ MLs, meanwhile the second process homogenized the WS_2_ on centimeter scale, increasing the thickness up to few layers as reported in (Rossi, 2018).

Monolayer graphene on sapphire was obtained via CVD on the c-plane of Al_2_O_3_(0001) substrates with a catalyst-free and single-step approach in a commercially available CVD reactor (HT-BM, Aixtron) (Mishra et al., 2019). Cleaned sapphire substrates were H_2_ etched in the HT-BM Aixtron reactor at 1180°C for 5 min in H_2_ atmosphere. After the H_2_-etching step, the substrates were annealed at 1200°C for 10 min in Ar atmosphere (1000 sccm). Then H_2_ (100 sccm) and CH_4_ (5 sccm) were introduced into the CVD system for 30 min at 25 mbar to grow graphene. Cool down was performed under argon flow (1000 sccm).

The controls adopted in the experiments were cleaned sapphire dices and polystyrene tissue-culture treated 48-well plates (Corning). The dimensions of all the substrates were about 6 mm × 6 mm.

Raman spectroscopy was performed with an InVia Renishaw with a 532 nm laser and a spot size of around 1 μm. AFM analysis was carried out with an AFM+ (Anasys Instruments) operated in tapping-mode.

Before cell culture, all substrates were sterilized by 30 min immersion in 96% ethanol and then rinsed several times with DI water.

### Preparation of hBDNF

The hproBDNF cDNA was subcloned in the prokaryotic expression vector pET11a and the protein was expressed as recombinant protein in E. coli strain BL21(DE3), refolded from inclusion bodies and purified by using protocols adapted from previously published ones (De Nadai et al., 2016; Di Matteo et al., 2017). Briefly, BL21(DE3) *Escherichia coli* were transformed with 30 ng of plasmid pET11a containing the gene of human proBDNF; bacteria were plated on Luria-Bertani (LB) agar plates supplemented with Ampicillin (Amp) and grown overnight at 37°C. Then a single colony was inoculated in 20 ml of LB supplemented with Amp and grown overnight at 37°C with shaking at 250 rpm. The day after, the inoculum was diluted 1:50 in 1L of LB + Amp and grown at 37°C at 250 rpm to reach an OD600 of about 1 before induction with 1 mM of isopropyl-b-thio-galactoside (IPTG). The proBDNF production was continued at 37°C at 250 rpm shaking. After 5 h bacteria were collected by centrifugation at 5000 × *g* for 10 min at 4°C. The bacterial pellet was first resuspended with Lysis Buffer (10 mM TRIS HCl pH 8, 1 mM EDTA and 1 mg/ml lysozyme) at 5 ml/g v/w ratio and incubated at room temperature for 1 h. After sonication on ice (3 cycles of 45 s on and 60 s off using a MicrosonTM Ultrasonic Cell Disruptor XL at maximum power), 3 mM MgCl_2_ and 50 μg/ml DNaseI solutions were added and the sample was incubated at room temperature for 30 min. To isolate inclusion bodies (IB), 0.5 vol. of Triton Buffer (60 mM EDTA, 1.5 M NaCl, 6% Triton X-100) was added and the sample was incubated at room temperature for 30 min on a stirring plate. The IB were then centrifuged at 18,000 × *g* for 30 min at 4°C, resuspended in 20 ml of resuspension buffer (10 mM TRIS HCl pH 8, 1 mM EDTA) 0.5 vol. of Triton Buffer and incubated at room temperature for 30 min on a stirring plate. The IB were then centrifuged at 18,000 × *g* for 30 min at 4°C and subsequently washed three times with a total volume of 100 ml of Washing Buffer (50 mM TRIS HCl pH 7.5, 1 mM EDTA). The IB pellet was then resuspended with 5 ml/g of Solubilization Buffer (6M guanidinium, 100 mM TRIS HCl pH 8, 1 mM EDTA and 100 mM DTT), and incubated until complete solubilization at room temperature on a rocking plate. Then the pH was lowered to 3.5 with HCl 3M and the sample was centrifuged at 18,000 × *g* for 30 min at 4°C. The resulting solution was dialysed three times against 300 ml of Dialysis Buffer (6M guanidinium pH 3.5) each one for 12 h using Visking dialysis membrane (cut-off 7 kDa, Medicell Membranes Ltd.) and the protein concentration was measured with the Bio-Rad Protein Assay. IB solution containing denatured hproBDNF was refolded by a drop by drop addition in 100 ml of Refolding buffer (1 M Arginin, 100 mM TRIS HCl pH 9.3, 5 mM EDTA pH 8, 1mM GSSG and 5 mM GSH). Every hour 50 μg/ml of sample were added to the buffer under vigorous stirring at 4°C. After 16–48 h the sample was dialyzed at 4°C using a Visking dialysis membrane (cut-off 12-14 kDa, Medicell Membranes Ltd.) against 2 L of IEX-A (50 mM Na phosphate pH 7, 1 mM EDTA) replacing the buffer after 12 h. The dialyzed sample containing proBDNF was filtered using a 0.22 μm filter and purified by FPLC. The sample was loaded on a HiLoad 16/10 SP Sepharose High Performance (∼20 ml – GE Healthcare) equilibrated with IEX-A buffer using a liquid chromatography system (ÄKTA^TM^ – FPLC). The protein was eluted with linear gradient from 0 to 100% of IEX-B (50 mM Na phosphate pH 7, 1 mM EDTA and 1M NaCl) at 1ml/min flow in 6 column volume (CV) and 2 ml fractions collected. The ones corresponding to the UV (280 nm) FPLC peak containing hproBDNF were pooled and dialyzed against 100 mM Hepes pH 7.5, 1 mM CaCl_2_. The mature hBDNF protein was obtained from the purified hproBDNF after digestion with previously prepared His-tagged human furin (1 μg enzyme : 20 μg of propeptide), at 30 °C for 2 h. The expression and purification of recombinant His-tagged human furin is reported below.

The hBDNF was purified by ion exchange chromatography with the same protocol used for the prohBDNF already described. The fractions collected were pooled and dialyzed against 1 L of Storage buffer (50 mM Na phosphate pH 7, 1 mM EDTA and 150 mM NaCl) for 16 h at 4°C. The dialyzed protein was concentrated up to 1mg/ml using an Amicon ultrafiltration membrane with 10 kDa cut-off (Merck-Millipore) and stored at −80°C. The purity and the correct molecular weight of the protein was monitored by SDS-PAGE and MS analyses.

### Expression and Purification of Recombinant His-Tagged Human Furin

The expression plasmid was kindly provided by Sven Dahms and the expression procedure adapted from Dahms et al. (2014) and Aricescu et al. (2006). Briefly, the coding sequence of human pro-furin, covering the amino acids 23–574, was inserted into the plasmid pHLsec. Using the restriction site AgeI, the insert was placed downstream to a secretion signal sequence encoded by the expression vector. The native protein sequence was modified with a Thrombin cleavage site and a His-tag, resulting in the artificial C-terminus SGSLVPRGSHHHHHH that is expressed after Ala574 (Aricescu et al., 2006; Dahms et al., 2014). Transfection and expression were performed in HEK293 cells. HEK293 cells were maintained in a humidified atmosphere at 37°C, 5% CO_2_ in Dulbecco’s Modified Eagle’s medium (DMEM high glucose, Gibco) supplemented with L-glutamine (Euroclone), non-essential amino-acids (Gibco), 10% fetal bovine serum (FBS;Euroclone) and 1% penicillin/streptomycin (Euroclone). Transfection was performed with Lipofectamine^®^ 2000 at the ratio (w/v) of plasmid-DNA to transfection reagent 1:5 (8.4 mg of plasmid-DNA and 42 ml of transfection reagent in 10-cm tissue culture dish). Transfection was carried out at approx. 80–90% confluence of the culture. The transfection medium was replaced after 4–5 h, lowering the serum concentration to 0.2%. Conditioned medium was harvested 72 and 120 h post transfection. 12 ml and 8 ml medium were used during the first and second culture period, respectively. The conditioned samples were centrifuged (20 min, 4500 *g*, 20°C), filtered using a PES membrane (0.22 mm pore size, Sarstedt) and stored at −20°C. Prior to purification the samples were thawed in ice, pooled and dialysed against a 20-fold excess of 25 mM Tris, pH 8,0, 250 mM NaCl, 5 mM CaCl_2_ with Visking dialysis membrane (cut-off 12-14 kDa, Medicell Membranes Ltd.) for 16 h at 4°C. His-tagged human furin was purified by affinity chromatography, using an ÄKTApurifier 100 FPLC system (GE Healthcare) with a 1 ml HisTrap FF Crude (GE-Healthcare) affinity column. Before purification, imidazole was added to the resulting samples, up to a concentration of 10 mM. The column was then washed with 10 CV of buffer A (100 mM Tris, pH 8.0, 500 mM NaCl, 5 mM CaCl_2_) supplemented with 10 mM imidazole. The protein was eluted with a linear gradient from 2 to 100% of buffer B (buffer A + 500 mM imidazole) at 1 ml/min flow in 20 CV and 2ml fractions collected. The ones corresponding to the UV (280 nm) FPLC peak were pooled and dialyzed against 10 mM Hepes, pH 7,5, 100 mM NaCl, 2 mM CaCl_2_ and then concentrated to 1 mg/ml, using an Amicon ultrafiltration membrane with 10 kDa cut-off (Merck-Millipore). The enrichment and the purity of the protein were monitored by SDS-PAGE analysis.

### SH−SY5Y Cell Culture and Differentiation

Human neuroblastoma SH−SY5Y cells (a kind gift of Prof. M. Canossa, University of Trento) were maintained in a humidified atmosphere at 37°C, 5% CO_2_ in high glucose Dulbecco’s modified eagle medium with nutrient mixture F12 (DMEM-F12) supplemented with 10% FBS and 1% penicillin/streptomycin (Gibco). Cell were plated at a density of 1 × 10^4^ cells/cm^2^ (∼40–60% confluency) onto the bare substrates. Differentiation was achieved using the following procedure: after 24 h from the seeding the cells were treated with 10 μM RA in high glucose DMEM-F12 supplemented with 1% penicillin/streptomycin and 10% of FBS for 5 days and then with hBDNF 100 ng/ml. Every 2–3 days, 2/3 of the medium was renewed.

The cells were observed at different time points using an inverted microscope equipped with a 20× magnification objective (Leica DMI4000B microscope). Typically, 10 fields per sample were acquired to perform morphometric analysis of SH-SY5Y differentiation. Three morphometric parameters were measured as previously reported in Marchetti et al. (2014): (i) the percentage of differentiated cells (Diff), determined counting in each field the number of cells with at least one neurite with a length equal to or longer than the cell body diameter, and expressed as percentage of the total number of cells in each field; (ii) the average number of neurites per cell in the field (av. neurites/cell), calculated analyzing more then 100 cells per condition; and (iii) the mean neurite length obtained measuring all the neurites of each differentiated cell in the field (length). The calculated values of Diff, Av. neurites/cell and Length are reported in Figure 3. The number of cells analyzed (nc) is the total pool of the experiments. The number of analyzed cells to calculate the mean neurite length was: DIV7, G *n* = 777, Sapphire *n* = 847, WS_2_ *n* = 757, Well *n* = 1057; DIV9, G *n* = 751, Sapphire *n* = 591, WS_2_ *n* = 499, Well *n* = 641.

At least 50 cells per fields were used for evaluating the percentage of differentiated cells. The number of analyzed cells was: DIV7, G *n* = 1872, Sapphire *n* = 1592, WS_2_ *n* = 2368, Well *n* = 1964; DIV9, G *n* = 1743, Sapphire *n* = 1970, WS_2_ *n* = 2693, Well *n* = 2484.

Cell viability was assessed with the Cell counting Kit-8 assay (Dojindo), based on quantification of WST-8 reduction due to the metabolic activity of viable cells. Samples were prepared according to the manufacturer’s instructions and measured at the GloMax Discover multiplate reader (Promega). The results are reported as % over the polystyrene well, considered as control. All the experiments were repeated at least twice independently.

### Statistical Analysis

For all the experiments, we performed two independent cultures with three to five biological replicates each. For length measurement and neurite number quantification, for each substrate we analyzed at least 200 cells from selected fields of the replicates obtained with a 20× objective. All data are expressed as the average value (mean) ± standard error of the mean (s.e.m.) unless stated otherwise. Data were analyzed by using Origin Software. After demonstrating the normality of our data with Shapiro-Wilk normality test, ANOVA with Bonferroni multiple comparison test was used for statistical significance with ^∗^*p* < 0.05, ^∗∗^*p* < 0.01, and ^∗∗∗^*p* < 0.001.

## Results and Discussion

### Characterization of WS_2_ and Graphene Grown by CVD on Sapphire

**FIGURE 1:**
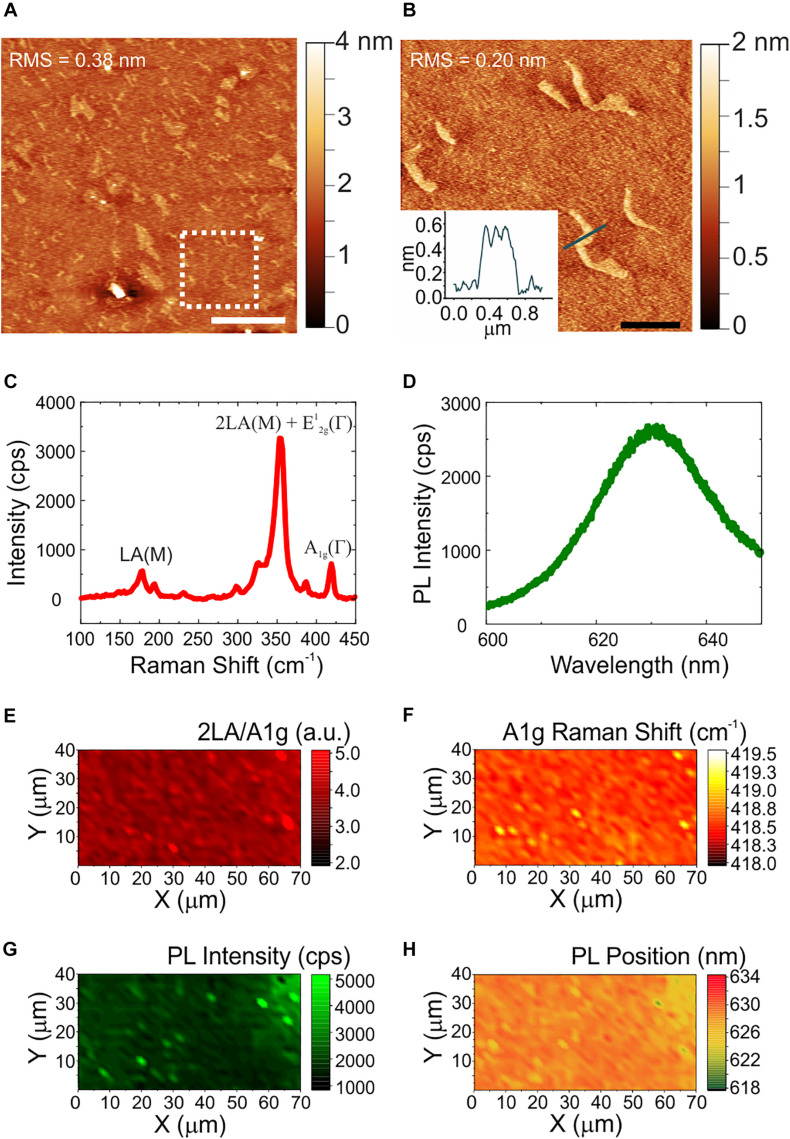
Morphological, structural and optical characterization of WS_2_. **(A)** Large area AFM topological map (scale bar: 5 μm). The dashed white square indicates the zoomed area reported in panel **(B)**, with a profile analysis of the patch (scale bar: 1 μm). The root mean square (RMS) values are reported on the maps. **(C,D)** Representative Raman and PL spectrum of few-layers WS_2_. **(E–H)** Maps of the ratio of the 2LA and A_1g_ modes, of the Raman shift of the A_1g_ mode, of the PL intensity, and of the PL peak position, respectively.

The quality, thickness and homogeneity of WS_2_ was analyzed using AFM and Raman spectroscopy. In this way, only the physicochemical effect of the material could be evaluated excluding any influence of inhomogeneities or topographical features of the samples. Figure 1 shows the morphological, structural and optical characterization of CVD WS_2_ on sapphire. Panel (a) reports a representative AFM topography image and a higher resolution micrograph with the relative line profile (Figure 1b). AFM analysis reveals the presence of some irregularly shaped patches of nanometric height. The height of these features is compatible to that of an additional WS_2_ layer (i.e., 0.7 nm). The overall root mean square (RMS) measured over an area of a 20 μm × 20 μm (5 μm × 5 μm) is 0.38 nm (0.20 nm), confirming the material flatness. The values are comparable to the ones obtained for the sapphire controls before hydrogen etching (Supplementary Figure 1), characterized by an atomically flat surfaces with some polishing scratches. Such variations in the nanometric scale are far away from the surface roughness that could influence neurons biological response (Brunetti et al., 2010). The WS_2_ number of layers and homogeneity over the entire sample was then assessed by Raman spectroscopy. A representative Raman spectrum collected on our WS_2_ samples is presented in Figure 1c. The Raman modes of WS_2_ appears at 178 cm^–1^ (LA(M) mode), 354 cm^–1^ (superimposed 2LA(M) and E^1^_2g_(Γ) modes) and 418.6 cm^–1^ (A_1g_(Γ) mode). It is worth noting that when a 532 nm laser is employed, there is the superimposition of the 2LA (second-order Raman resonance involving the longitudinal acoustic mode (LA)) mode with the E_2g_ Raman mode (Berkdemir et al., 2013), so it is not possible to assess WS_2_ thickness using the separation of the E_2g_ and A_1g_ modes. A benchmarking method for WS_2_ monolayer is that the ratio of the 2LA(M) and A_1g_(Γ) Raman modes is higher than 2.2, as previously demonstrated in Berkdemir et al. (2013). Also, the Raman shift of the A_1g_(Γ) peak can be used. In case of WS_2_ monolayer the A_1g_(Γ) occurs at 417 cm^–1^ and the shift toward higher Raman shift is indicative of few-layer up to 420 cm^–1^, that is the average value for bulk WS_2_ (Berkdemir et al., 2013). For our samples, the ratio of the 2LA(M) and A_1g_(Γ) is 4.2, compatible with monolayer thickness. The position of the A_1g_(Γ) is 418.5 cm^–1^, suggesting bilayer WS_2_ (Berkdemir et al., 2013). By evaluating WS_2_ photoluminescence (PL) (see panel d) we find a broad, faint peak at 630 nm (1.97 eV) redshifted of 12 nm with respect to the standard PL emission of monolayer WS_2_ on insulating substrates (Park et al., 2015). Hence, we conclude that our WS_2_ is mostly bilayer. The thickness homogeneity of the materials is confirmed in panels (e)-(h) that show the maps of the 2LA(M)/A_1g_(Γ) intensity ratio, of the A_1g_(Γ) Raman shift, of PL intensity and peak position, respectively. The A_1g_(Γ) mode Raman shift in panel (f) is consistently found to be at an average position of 418.5 cm^–1^. Only in some areas with lateral length of about 1 micrometer, A_1g_(Γ) is locally peaked at 419 cm^–1^ and the PL peak is higher and blueshifted [see panels (f–h)], confirming locally the presence of an additional WS_2_ layer as already visualized by AFM. Further statistical analysis of the Raman and PL data is reported in the Supplementary Material (Supplementary Figure 2).

**FIGURE 2:**
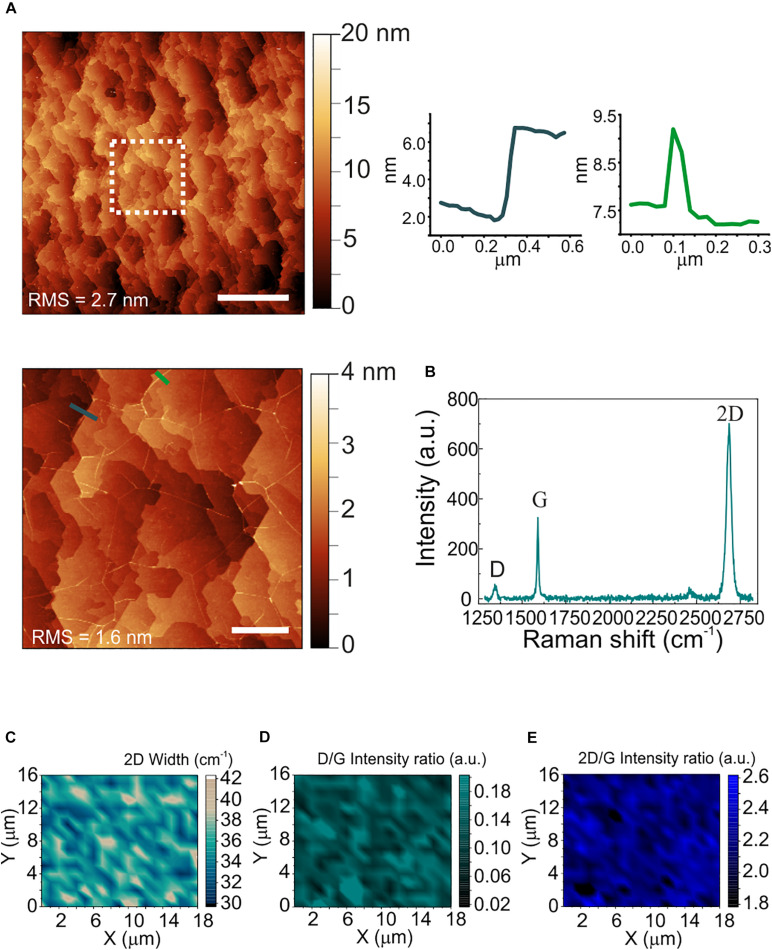
Morphological and structural characterization of polycrystalline graphene on sapphire. **(A)** Large area AFM topological map (scale bar: 5 μm). The dashed white square indicates the zoomed area reported on the bottom. Right: line profiles across an atomic step **(left)** and a ridge **(right)** (scale bar: 1 μm). The RMS values are reported on the maps. **(B)** Representative Raman spectrum of polycrystalline graphene grown on sapphire. The 2D width is 34 cm^–1^, the D/G and the 2D/G intensity ratios are 0.18 and 2.1, respectively. **(C–E)** Raman maps of the 2D mode width, the D/G intensity ratio and the 2D/G intensity ratio, respectively.

The morphological and structural characterization of graphene on sapphire is reported in Figure 2. Differently from WS_2_, a sapphire surface treatment with hydrogen was necessary before graphene growth, to improve the graphene crystalline quality (Mishra et al., 2019). The AFM micrograph in panel (a) shows the atomic terraces of the sapphire substrate which become visible after hydrogen etching and graphene growth (Mishra et al., 2019). Step heights are integer multiples of the single unit cell height (i.e., 1.3 nm), and graphene ridges (3–4 nm high) – which are due to different thermal expansion coefficient of graphene and sapphire – are visible. The RMS roughness measured over an area of 20 μm × 20 μm is 2.7 nm. The increased roughness with respect to the WS_2_ and sapphire substrates is ascribed to the atomic terraces, originated by the sapphire hydrogen etching more than graphene growth (Mishra et al., 2019). A representative Raman spectrum of graphene grown on sapphire is reported in Figure 2b. The standard graphene G and 2D modes appear at 1589 cm^–1^ and at 2684 cm^–1^, respectively, while the defects-activated D mode appears at 1345 cm^–1^. Maps of the 2D width, of the D/G and of the 2D/G intensity ratios are respectively employed to spatially assess strain and doping fluctuations, defect concentration and graphene doping. The 2D full width at half maximum (FWHM; Figure 2c) shows an average value of 35 cm^–1^, comparable with those previously reported for epitaxial and CVD graphene used as cell culture platform (Convertino et al., 2018, 2020). The D/G and 2D/G intensity ratios indicate a low defect density and a carrier concentration of around 4 x 10^12^ cm^–2^ (See Supplementary Figure 3 for more details). The average values of the 2D FWHM and the 2D/G peak intensity ratio confirm that graphene is monolayer (Mishra et al., 2019).

### Cellular Viability and Differentiation of SH-SY5Y on CVD WS_2_

**FIGURE 3:**
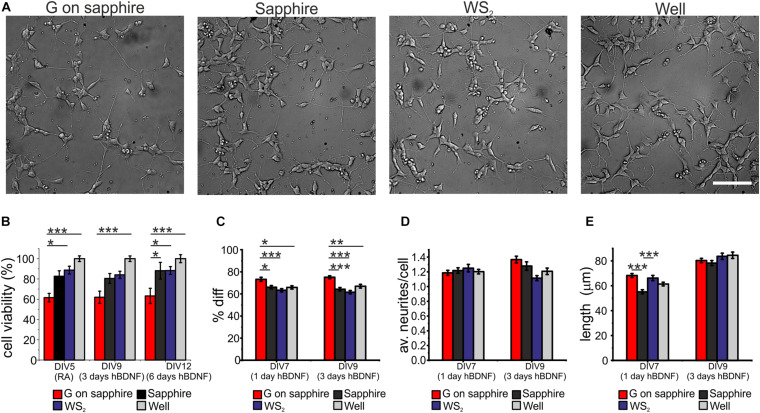
SH-SY5Y cells cultured on WS_2_, graphene grown on sapphire and control substrates. **(A)** Typical optical microimages of SH-SY5Y cells grown on graphene on sapphire (G on sapphire), sapphire, WS_2_ and polystyrene (well) at day 9, after 3 days of hBDNF treatment (scale bar: 100μm). **(B)** Cell viability after 5, 9, and 12 days tested by WST-8. The results are reported as % over the polystyrene control sample. Histograms show **(C)** quantification of the percentage of differentiation, **(D)** average number of neurites per cell and **(E)** neurite length at different DIV of two independent experiments per substrate. Data reported as mean ± standard error of the mean (s.e.m.). Nonparametric Kruskal–Wallis test was used for statistical significance, with *p < 0.05, **p < 0.01, ***p < 0.001.

We investigated the cytotoxicity of WS_2_ and compared it to that of graphene, the sapphire substrate and control wells, by morphological observation of differentiated SH-SY5Y and WST-8 viability assay. Results for cell differentiation according to the RA/hBDNF differentiation protocol used to have neuron-like cells are shown in Figure 3A. At day 3 of differentiation with hBDNF there were no morphological variations between the substrates. As visible in panel (a) cells adhered uniformly to the underlying substrates and they were all characterized by neural morphology with significant neurite outgrowth. The cell viability was assessed at three different time points: at DIV5 (the last day of RA treatment before hBDNF treatment), DIV7 (day 1 of hBDNF treatment) and DIV9 (day 3 of hBDNF treatment) (Figure 3B). Cell adhered on sapphire even in the absence of a coating in agreement with previous reports (Khan et al., 2011). As visible in panel (b), we did not observe significant differences in cell viability between WS_2_ (DIV5: 88.7 ± 3.8%, DIV9: 84.1 ± 3.5%, DIV12: 88.3 ± 3.9%) and sapphire (DIV5: 82.7 ± 5.3%, DIV9: 80.5 ± 4.8%, DIV12: 88.1 ± 8.2%), that showed a signal of about 80% over the polystyrene control wells in all the probed times. This confirm previous results obtained on WS_2_ film deposited by the drop-casting method on glass (Suhito et al., 2017) and on mechanical exfoliated WS_2_ (up to 10 μm in diameter) on PDMS (Appel et al., 2016), where a surviving rate comparable to the control was demonstrated for mesenchymal and epithelial cells, respectively. Mammalian cells were also cultured on CVD WS_2_ film transferred on PDMS for morphometric analysis, but no detailed results on cell viability were reported (Palumbo et al., 2018). On the contrary, a significantly reduced viability was observed for graphene (DIV5: 61.6 ± 4.2%, DIV9: 62.0 ± 6.0%, DIV12: 63.2 ± 7.5%) with respect to the control well (^∗∗∗^*p* < 0.001), sapphire (DIV12: ^∗^*p* < 0.05) and WS_2_ (DIV5: ^∗^*p* < 0.05; DIV12: ^∗^*p* < 0.05). It is not clear what causes the reduced cell viability on graphene, probably it depends on an intrinsic difference in surface chemistry (Lee et al., 2015). The surfaces roughness for all the substrates was in the same order of magnitude (see Supplementary Material). In addition, the presence of the nanometric steps should not influence cell viability, since epitaxial graphene on silicon carbide (SiC), characterized by the same morphology with atomic terraces, was showed to have a comparable viability to the other control substrates, including hydrogen etched SiC (Convertino et al., 2018). Moreover, the contact angle measurements revealed comparable values for graphene and WS_2_, that were found to be more hydrophobic than sapphire (^∗∗∗^*p* < 0.001 and ^∗^*p* < 0.05, respectively) (Supplementary Figure 4), as showed for graphene on SiC (Convertino et al., 2018). For these reasons, we excluded both topography and wettability. We tested if the polymeric coating could improve the cell viability, but no statistically significant differences between bare and coated substrates was observed (Supplementary Figure 5). We speculate that the reduced number of cells on graphene after the coating can be due to the inhomogeneous collagen distribution on graphene, described in our previous work (Convertino et al., 2018).

The morphometric analysis of the differentiation process was performed at day 1 and day 3 of hBDNF treatment. We quantified three morphometric parameters: the percentage of differentiated cells in each field (Diff) reported in panel (c), the average neurites number per cell (av. neurites/cell) reported in panel (d) and the average neurites length of differentiated cell (length) reported in panel (e). More than 60% of the cells differentiate on sapphire (1 day hBDNF: 66.1 ± 1.5%, 3 days hBDNF: 64.2 ± 1.5%), WS_2_ (1 day hBDNF: 63.3 ± 1.4%, 3 days hBDNF: 61.5 ± 1.5) and control well (1 day hBDNF: 65.9 ± 1.5%, 3 days hBDNF: 67.0 ± 1.7%), showing that WS_2_ did not affect cell differentiation. On the contrary, we found that graphene (1 day hBDNF: 73.2 ± 1.9%, 3 days hBDNF: 75.1 ± 1.3%) induced significantly greater differentiation relative to the control well, both after one (^∗^*p* < 0.05) and three days (^∗∗^*p* < 0.01) of hBDNF treatment (Figure 3C). An improved differentiation on graphene was observed also when compared to sapphire (^∗^*p* < 0.05 after one day and ^∗∗∗^*p* < 0.001 after three days) and WS_2_ (^∗∗∗^*p* < 0.001 both after one and three days). Remarkably, after one day of hBDNF treatment the percentage of differentiated cells on graphene was higher than on WS_2_, sapphire and well by 11, 16 and 11%, respectively, reaching an increase in the percentage of 17, 22, 12% after three days of hBDNF. The increased differentiation confirmed previous results reported for different neural cells and ascribed to the material’s electrical conductivity, known to affect neuronal differentiation (Agarwal et al., 2010; Li et al., 2011; Park et al., 2011; Defteralı et al., 2016; Convertino et al., 2018). Moreover, knowing that proliferation rate decreases in differentiated SH-SY5Y cells in comparison to the undifferentiated one, the cell proliferation and thus the viability assay could be influenced by this different differentiation (Kovalevich and Langford, 2013). The average number of neurites per cell on WS_2_ (1 day hBDNF: 1.25 ± 0.05, 3 days hBDNF: 1.11 ± 0.03) was comparable to that of the controls (sapphire: 1 day hBDNF: 1.22 ± 0.04, 3 days hBDNF: 1.28 ± 0.06; graphene: 1 day hBDNF: 1.19 ± 0.03, 3 days hBDNF: 1.37 ± 0.05; control well: 1 day hBDNF: 1.20 ± 0.03, 3 days hBDNF: 1.21 ± 0.04) (Figure 3D). We evaluated the mean neurite length at day 1 and day3 of hBDNF treatment (Figure 3E). Following this time, the formation of a highly dense network impeded further quantification. Interestingly, after one day of differentiation the mean neurite length on WS_2_ (66.2 ± 2.1 μm) and graphene (68.2 ± 1.6 μm) was significantly higher than on sapphire (55.1 ± 1.6 μm) (^∗∗∗^*p* < 0.001) by 20 and 24%, respectively, resembling the percentage increase observed for epitaxial graphene on SiC with respect to control substrate (Convertino et al., 2018). After 3 days of hBDNF treatment, the neurite length on WS_2_ (83.7 ± 2.5 μm) and graphene (80.3 ± 1.7 μm) was comparable with the control (sapphire: 78.2 ± 2.0 μm and control well: 84.5 ± 2.6 μm) (panel (e)). The reduced neurite elongation in a more mature culture can be ascribed to a underestimation of the real neurite length, due to the increased neurite network complexity with the time or to a phase of adaptation experienced by the cell, as described for primary neurons on CVD graphene (Convertino et al., 2020).

These results indicate that WS_2_ did not affect cell viability and differentiation, showing values comparable with the ones of the control wells. Interestingly, WS_2_ induced also a neurite sprouting comparable to the other substrates, better than bare sapphire, confirming its potential in biomedical application and interactions with neural cells. Graphene confirmed its positive effect on cell differentiation and neurite outgrowth, as previously reported in different cell types including SH-SY5Y (Li et al., 2011; Lee et al., 2012; Convertino et al., 2018, 2020). In order to investigate WS_2_ influence in a more complex model that better reflects the in vivo environment, further studies are necessary using primary neurons. Besides the replica of the key findings reported in this work, the use of primary neurons can help to investigate the mechanisms driving axon elongation on this material, opening new routes for axon regeneration applications. The effect of the material on the neuronal network electrical activity can be explored with electrophysiological measurements, which will ultimately help to understand the material effect on network formation and neuron excitation, to correlate this finding with axon elongation (Convertino et al., 2020).

## Conclusion

This work provides novel insights about the use of large-scale WS_2_ grown via CVD on a transparent biocompatible substrate as an attractive platform for cell culture. We used SH-SY5Y cells, as a consolidated model for neurological studies to examine the substrate effect on cell morphology and viability. The results were compared with standard tissue culture wells, the sapphire substrate, and with graphene grown on sapphire, being the latter the most studied 2D materials with potential as neural interface. Interestingly, WS_2_ appears to be an excellent substrate for culturing SH-SY5Y cells, showing a neurite length and viability comparable to the control well and an increased neurite length (66.2 ± 1.6 μm, 20% longer with respect to sapphire, ^∗∗∗^*p* < 0.001). Graphene confirmed its capacity to induce cell differentiation and neurite sprouting, performing better than controls.

## Data Availability Statement

The original contributions presented in the study are included in the article/Supplementary Materials, further inquiries can be directed to the corresponding author/s.

## Author Contributions

DC, LM, FF, and CC conceptualized the study. NM, DC, FF, and CC synthesized and characterized graphene substrates. FF, DC, and CC synthesized and characterized WS_2_ substrates. MC, AV, and AC performed the preparation of hBDNF and expression and purification of recombinant His-tagged human furin. DC and LM established SH-SY5Y cultures on WS_2_ and graphene and performed differentiation and cell viability experiments and data analysis. DC, LM, FF, and CC wrote the manuscript with contributions from all authors.

## Conflict of Interest

The authors declare that the research was conducted in the absence of any commercial or financial relationships that could be construed as a potential conflict of interest.
