# Supplementary material for: Effect of Chemical Vapor Deposition WS2 on Viability and Differentiation of SH-SY5Y Cells
Source: Front Neurosci. 2020 Oct 30;14:592502. doi: 10.3389/fnins.2020.592502 (PMC7662391; doi:10.3389/fnins.2020.592502)
Supplement: Supplementary file 1 [file Image_1.pdf]

Supplementary Material

Effect of chemical vapor deposition WS_2 on viability and differentiation of SH-SY5Y cells

D. Convertino, N. Mishra, L. Marchetti, M. Calvello, A. Viegi, A. Cattaneo, F. Fabbri and C. Coletti

1 Supplementary results on sapphire and culture well controls AFM characterization

AFM analyses of sapphire and polystyrene culture well evidence a different surface topography. The sapphire surface is atomically flat. Some scratches originating from the substrate polishing process are also visible. The overall surface roughness was highest in the polystyrene well, due to the presence of numerous scratches.

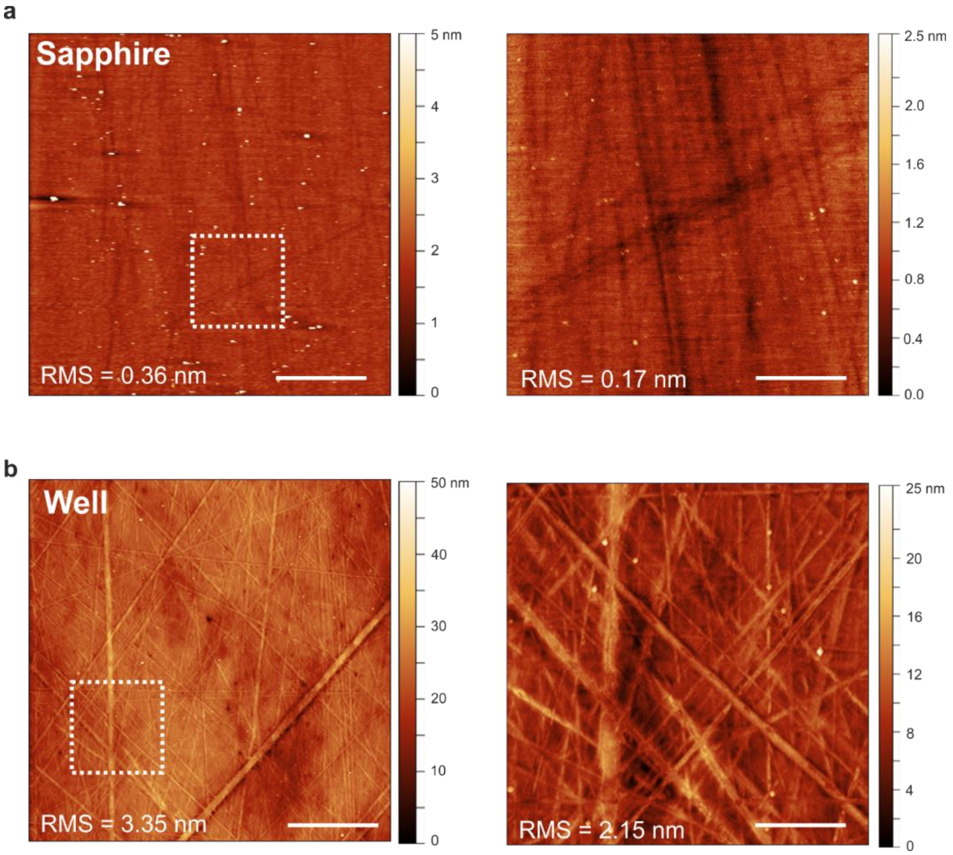

Figure S1: (a,b) Large area AFM topological map of sapphire (a) and culture well (b) (scale bar: 4 µm). The dashed white square indicates the zoomed area reported on the right (scale bar: 1 µm).

2 Supplementary results on WS_2 Raman and PL characterization

The 2LA(M) / $A_{1g}(\Gamma)$ intensity ratio map in Figure 1 in the main text is rather homogeneous. The data of the map, resumed in a histogram in Figure S2(a), shows that the distribution is peaked at 3.8 with a full width half maximum of 1, therefore the 2LA(M) / $A_{1g}(\Gamma)$ intensity ratio never goes below the 2.2 value, these data clearly demonstrate that the 2LA(M) / $A_{1g}(\Gamma)$ intensity ratio method is rather inaccurate compared to the $A_{1g}(\Gamma)$ method, probably due to the superimposition of the $A_{1g}(\Gamma)$ with the sapphire substrate mode. Indeed, the histogram of the $A_{1g}(\Gamma)$ Raman shift in panel (b) reports an average position 418.5 cm^{-1} with a FWHM of 0.3 cm^{-1}, in agreement with the previous assessment of a WS_2 bilayer.

The histograms of Figure S2 (c) and (d) show the distribution of the PL intensity and peak position of Figure 1(g) and (h) in the main text. Both the statistical distributions have a tail with respect to the main peak, due to the presence in the maps of bright spots with a higher intensity and a blue-shifted PL peak position. In case of the PL intensity the average value is 1900 cps with a FWHM of 700 cps, with a tail on the high intensity side up to 4000 cps. Meanwhile the PL peak position distribution is peaked at 629 nm with a FWHM of 2 nm and a tail on the low wavelength side down to 625 nm. These values further confirm the presence of trilayer patches, as reported in the main text.

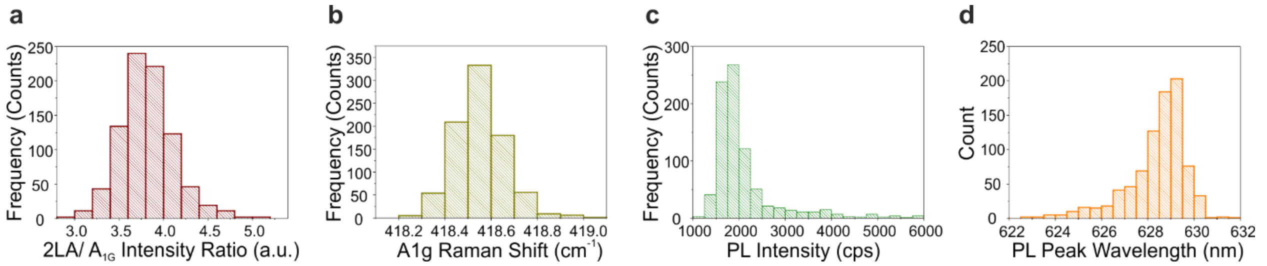

Figure S2: Statistical representation of the Raman map data for (a) the ratio of the 2LA and A1g modes, (b) the A1g Raman shift, (c) the PL intensity and (d) the PL peak position.

3 Supplementary results on Raman characterization of graphene on sapphire

Figure S3 show the histograms for the maps presented in Figure 2 (c-e). The 2D width histogram in panel (a) shows an average value of 35 cm^{-1} with a FWHM of the distribution of 6 cm^{-1}. The D/G intensity ratio has the average value at 0.12 with a FWHM of 0.1. The doping extracted from the average value (2.1) of the 2D/G intensity ratio histogram is 4x 10^{12} cm^{-2}.

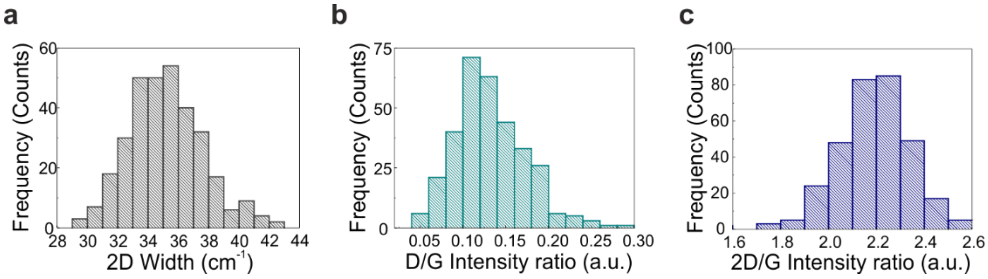

Figure S3:.Statistical representation of the map data for (a) the 2D mode width, (b) the D/G intensity ratio and (c) the 2D/G intensity ratio.

4 Supplementary results on substrate wettability

Non coated substrates showed a different wettability. Sapphire was significantly more hydrophilic than graphene on sapphire (***$p < 0.001$), WS_2 (*$p < 0.05$) and polystyrene culture well (***$p < 0.001$), while graphene on sapphire, WS_2 and the control well had comparable contact angle values. Contact angles were measured using a CAM 101 contact angle meter, from KSV Instruments Ltd. (Finland) and estimated by measuring the angles between the baseline of the droplet and the tangent at the droplet boundary.

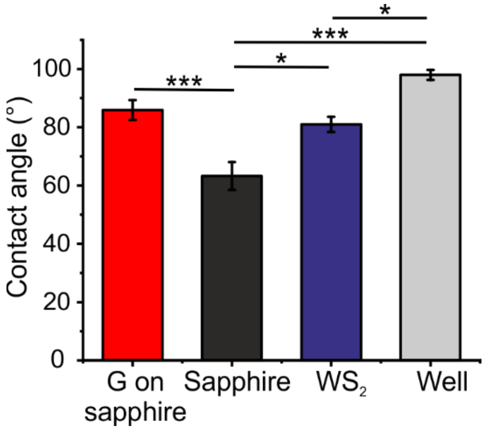

Figure S4: Contact angle measurements of WS_2, graphene grown on sapphire and control substrates. Values are the mean ± s.e.m.. All measurements were made using DI water as a probe liquid. For each substrate we analyze n samples (G on sapphire: n = 8, Sapphire: n = 11, WS_2: n = 8, Well: n = 13).

5 Coating vs no coating

We compared the coating effect on cell viability. Cell were plated at ~40–60% confluency onto the substrates previously coated with 100μg/ml collagen type I solution in water (C3867 Sigma-Aldrich) for 4 h at 37 °C and then washed two times in DI water. Bare substrates (the same used in the morphological quantification in the main text) were used as comparison. Cell viability after 5, 9, and 12 days, corresponding at 1 day treatment with RA, 3 days treatment with hBDNF and 6 days treatment with hBDNF was tested by WST-8. No statistically significant differences were observed between bare and coated substrates of each type.

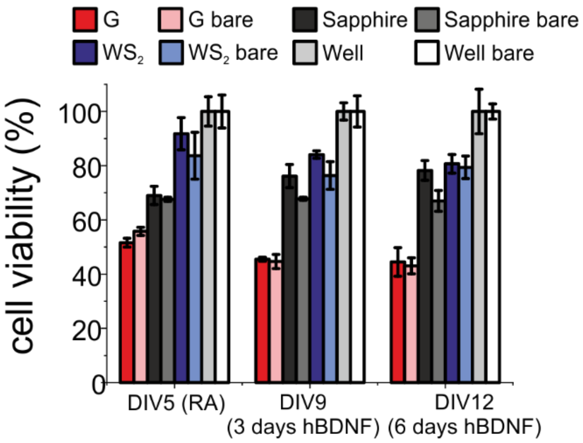

Figure S5: Cell viability on bare and coated substrates. Cell viability after 5, 9, and 12 days tested by WST-8. The results are reported as % over the bare polystyrene control sample. Data reported as mean ± s.e.m.. The cell viability on bare and coated substrates did not significantly differ, according to Nonparametric Kruskal–Wallis test.
